# Savvy Seniors: A Content Analysis of the Usability of Older Adult Resource Websites

**DOI:** 10.1093/geroni/igaa057.1330

**Published:** 2020-12-16

**Authors:** Kendall Weber, Deborah Koh, Lisa Stone, Andrew Lac

**Affiliations:** 1 University of Colorado at Colorado Springs, Colorado Springs, Colorado, United States; 2 University of Colorado Colorado Springs, Colorado Springs, Colorado, United States

## Abstract

Due to age-related declines, older adults often experience difficulties using the Internet and navigating websites, even among websites specifically designed for them. Subsequently, the National Institute on Aging (NIA) and National Library of Medicine (NLM; 2001) published guidelines to promote website usability among a later life population. Applying quantitative content analysis, this study examined the accessibility of senior resource websites based on NIA and NLM recommendations. Specifically, the relation between the type of organization sponsoring the websites (i.e., governmental, non-profit, or private) and overall accessibility and the relation between the number of resource categories (e.g., housing, financial, etc.) and overall accessibility were investigated. Font size, organization of presented information, and the color and contrast on the websites were coded to determine how they contribute to overall accessibility. Using a sample of the 100 most-searched senior resource websites, a one-way ANOVA indicated that the type of site on overall accessibility was not significant, F(2, 97) = 2.29, p = .11, η2 = .04. The multiple regression model showed that font size (β = .30, p < .001), organization of information (β = .38, p < .001), and color and contrast (β = .45, p < .001) were significant predictors of overall website accessibility and explained 62% of the variance, F(3, 96) = 51.18, p < .001. The findings suggest that various NIA guidelines differentially contribute to a website’s accessibility, and further attention should be paid to cognitive aspects of resource websites.

